# Expansion of HAART Coverage Is Associated with Sustained Decreases in HIV/AIDS Morbidity, Mortality and HIV Transmission: The “HIV Treatment as Prevention” Experience in a Canadian Setting

**DOI:** 10.1371/journal.pone.0087872

**Published:** 2014-02-12

**Authors:** Julio S.G. Montaner, Viviane D. Lima, P. Richard Harrigan, Lillian Lourenço, Benita Yip, Bohdan Nosyk, Evan Wood, Thomas Kerr, Kate Shannon, David Moore, Robert S. Hogg, Rolando Barrios, Mark Gilbert, Mel Krajden, Reka Gustafson, Patricia Daly, Perry Kendall

**Affiliations:** 1 BC Centre for Excellence in HIV/AIDS, Providence Health Care, Vancouver, British Columbia, Canada; 2 Division of AIDS, Department of Medicine, University of British Columbia, Vancouver, British Columbia, Canada; 3 Faculty of Health Sciences, Simon Fraser University, Vancouver, British Columbia, Canada; 4 BC Centre for Disease Control, Vancouver, British Columbia, Canada; 5 Vancouver Coastal Health Authority, Vancouver, British Columbia, Canada; 6 Ministry of Health, Province of British Columbia, Victoria, British Columbia, Canada; University of Pittsburgh, United States of America

## Abstract

**Background:**

There has been renewed call for the global expansion of highly active antiretroviral therapy (HAART) under the framework of HIV treatment as prevention (TasP). However, population-level sustainability of this strategy has not been characterized.

**Methods:**

We used population-level longitudinal data from province-wide registries including plasma viral load, CD4 count, drug resistance, HAART use, HIV diagnoses, AIDS incidence, and HIV-related mortality. We fitted two Poisson regression models over the study period, to relate estimated HIV incidence and the number of individuals on HAART and the percentage of virologically suppressed individuals.

**Results:**

HAART coverage, median pre-HAART CD4 count, and HAART adherence increased over time and were associated with increasing virological suppression and decreasing drug resistance. AIDS incidence decreased from 6.9 to 1.4 per 100,000 population (80% decrease, p = 0.0330) and HIV-related mortality decreased from 6.5 to 1.3 per 100,000 population (80% decrease, p = 0.0115). New HIV diagnoses declined from 702 to 238 cases (66% decrease; p = 0.0004) with a consequent estimated decline in HIV incident cases from 632 to 368 cases per year (42% decrease; p = 0.0003). Finally, our models suggested that for each increase of 100 individuals on HAART, the estimated HIV incidence decreased 1.2% and for every 1% increase in the number of individuals suppressed on HAART, the estimated HIV incidence also decreased by 1%.

**Conclusions:**

Our results show that HAART expansion between 1996 and 2012 in BC was associated with a sustained and profound population-level decrease in morbidity, mortality and HIV transmission. Our findings support the long-term effectiveness and sustainability of HIV treatment as prevention within an adequately resourced environment with no financial barriers to diagnosis, medical care or antiretroviral drugs. The 2013 Consolidated World Health Organization Antiretroviral Therapy Guidelines offer a unique opportunity to further evaluate TasP in other settings, particularly within generalized epidemics, and resource-limited setting, as advocated by UNAIDS.

## Introduction

After 30 years, controlling the HIV epidemic remains an extraordinary challenge. This is despite the availability of a number of proven prevention tools, including harm-reduction strategies and emerging biomedical interventions. [1]–[7] Recently, increasing attention has been focused on the potential role that the expansion of HIV treatment may offer to curb progression to AIDS and premature death among HIV infected individuals and secondarily reduce HIV transmission, commonly referred to as HIV treatment as prevention or TasP. [8]–[11] In brief, HIV-1 RNA concentration (hereafter referred to as viral load) is a key determinant of the level of risk associated with sexual, vertical and needle sharing-related HIV transmission. [12]–[21] Appropriate use of HAART suppresses HIV replication on a sustained basis, leading typically to undetectable, viral load in plasma and halting disease progression to AIDS and premature death.^11^ In addition, as viral load rapidly declines in plasma and subsequently in other biological fluids (including semen, vaginal fluids and rectal mucosa), the likelihood of HIV transmission per exposure event is markedly reduced. [12]–[21] The concept of scaling up highly active antiretroviral therapy (HAART), commonly referred to as TasP, has gained substantial momentum, as its efficacy and effectiveness have become increasingly apparent. [8], [9], [22]–[24] However, the real-world population-level effectiveness and sustainability of this strategy remains to be adequately characterized.

British Columbia (BC), Canada, provides a unique environment to address this issue within a concentrated HIV epidemic. HAART eligibility in BC has remained consistent with the IAS-USA guidelines since 1996 to the present. [11], [25] BC is an adequately resourced environment with a publically funded health care system, that fully subsidizes access to medical services, centralized laboratory monitoring and access to HAART with no co-payments or deductibles. In addition, a single agency, the BC Centre for Excellence in HIV/AIDS (BC-CfE), is responsible for the centralized distribution of all antiretrovirals and monitoring of key HIV-related outcomes in BC. Additionally, the availability of unique personal health numbers for all BC-residents provide unique opportunities to evaluate the impact of this strategy throughout the province using anonymized data linkages between administrative datasets.

We therefore conducted a longitudinal ecological study to evaluate the population-level effectiveness and sustainability of HAART expansion in BC. Specifically, we sought to characterize the association between HAART coverage, and the proportion of individuals virologically suppressed with the number of new AIDS diagnoses, and all-cause mortality among HIV-infected BC residents, as well as the number of new HIV diagnoses and estimated HIV incident cases between 1996 and 2012.

## Methods

Aggregate- and individual-level data on the key study variables were collected from a number of sources, including the BC-CfE Laboratory and Drug Treatment Registries, the BC Centre for Disease Control (BCCDC), the BC Ministry of Health (BC MoH) population-level health resource utilization files, and the Public Health Agency of Canada (PHAC).

### HIV Incidence and Prevalence

We also obtained HIV prevalence estimates for BC from 1996 to 2011, independently generated by the Public Health Agency of Canada (PHAC) [26] using previously published methods. [27] The method is a modified back-projection method to estimate HIV incidence and prevalence. Unlike the back-projection methods used the literature, this new method does not require linking HIV and AIDS diagnostic registries. It is based on linking the estimated parametric distribution between the time to HIV testing and time since HIV infection. These distributions were adjusted for testing practices over time (e.g. HIV testing trends), reporting delays, multiple reporting of cases, AIDS cases reporting (adjusting for the effect of HAART), survival time before and after HAART was introduced, and birth cohort effects. [28], [29] Data for 2012 are not yet available, and therefore, we extrapolated the available data to obtain preliminary estimates for these two indicators for the year 2012.

### New HIV Diagnoses and AIDS Morbidity and Mortality

The BCCDC collates all BC HIV surveillance data and houses HIV testing data from the provincial public health reference laboratory, which accounts for approximately 90% of all HIV screening and all confirmatory testing in BC. Of note, HIV infection became provincially reportable in 2003.^30^ Data regarding new HIV diagnoses were extracted from annual and monthly BCCDC HIV/AIDS Update and Reportable Diseases reports from 1996 to 2012. [30] The AIDS case-reports were allocated according to the year when a client was diagnosed with the first AIDS defining illness.

### HIV Treatment and Monitoring

HIV/AIDS care is fully publically funded by the health care system in BC. This includes publically funded access to medical services, virological and laboratory monitoring and HAART (with no co-payments or deductibles). HAART use in BC is driven by the BC-CfE HIV treatment guidelines, [31] which have remained consistent with those of the International AIDS Society-USA (IAS-USA) since 1996. [11], [25].

Clinical, treatment and laboratory data were obtained from the BC-CfE, which has a centralized system capturing all antiretroviral distribution, all plasma viral load testing, and all resistance testing, as well as baseline CD4 count for 96% of all patients starting antiretroviral therapy in BC. Specifically, HAART coverage, CD4 cell counts, viral load, treatment adherence (as measured by validated prescription refill compliance), [32] and genotypic drug resistance were obtained from the BC-CfE databases. For HAART coverage, we obtained the yearly number of individuals on HAART in BC. Viral Load data were adjusted for changes in viral load assay sensitivity over time, as previously described. [33] For the purpose of these analyses, we recorded the highest viral load for every individual per year. To accommodate irregular frequency of measurements or missing values, the highest yearly value was carried forward until a new measurement was available. Individuals were censored if they moved out of the province or died.

Population-level adherence to antiretroviral therapy was assessed by prescription refill compliance for each year as previously described. [32] In brief, this was estimated by dividing the number of months of medications dispensed by the number of months of follow-up during each calendar year. Resistance testing was performed on stored viral load samples collected immediately before starting HAART and upon virological rebound thereafter. Samples were assigned to 1 of 4 resistance categories on the basis of a modified International AIDS Society-USA table, as previously described. [34] In brief, samples were considered to be resistant if they displayed one or more major resistance mutations in any of the following 4 categories: I) lamivudine, or emtracitabine; II) other nucleoside reverse transcriptase inhibitors; III) protease inhibitors; and IV) non-nucleoside reverse-transcriptase inhibitors. Then, the data were classified into the following categories: I) individuals with no evidence of HIV resistance or with wild type HIV only II) those who were never genotyped, and individuals demonstrating HIV resistance to III) one, IV) two or V) three antiretroviral drug classes, respectively. Those who were never genotyped include individuals with viral load samples <250 copies/mL, which were not tested given that sequence analysis is not reliable in this setting. Antiretroviral resistance data are displayed by calendar year, and include all individuals who have ever enrolled on the BC-CfE program - whether or not they ever used antiretrovirals, from January 1996 to December 2012.

### All-Cause Mortality among HIV-Positive Individuals

HIV-related mortality data were obtained from BC Vital Statistics for all HIV-positive individuals regardless of whether or not they had or not ever accessed antiretroviral therapy between 1996 and 2011. [35] Data for 2012 were not yet available. The HIV-related diagnoses were based on the 10^th^ revision of the International Statistical Classification of Diseases and Related Health Problems (ICD-10) codes B20 to B24. [36].

### Population Data

Population estimates were obtained for the years 1996–2011 to calculate mortality and AIDS rates. These estimates were obtained through Statistics Canada through BCStats. [37] Data for 2012 were not yet available.

### Statistical Analysis

To be consistent throughout the text, we included the year 2012 for all variables. However, since the estimates of the Public Health Agency of Canada were complete until 2011, we used a polynomial function of degree three to extrapolate the data. This polynomial function provided the best fit for the incidence and prevalence data from 1996 to 2011, and we therefore used estimated values of these two indicators for 2012.

We modeled trends using generalized additive models, which accounts for the non-linear temporal trends in these longitudinal data. [38], [39] We fitted different Poisson regressions, [40] with correction for over dispersion in the data, for each of the following outcomes: estimated HIV incidence rate, HIV-related mortality rate and the rate of AIDS cases. The primary explanatory variables of interest were the number of individuals actively on HAART and the percentage of individuals with the highest yearly viral load lower than 500 copies/mL. Note that for the rates, the size of the BC population was used as an offset in the models. These models were performed using the statistical package SAS (version 9.3). All p values reported were two-sided, and significance was set at the 5% level.

### Ethics Approval

The BC-CFE received approval for this study from the University of British Columbia ethics review committee at the St Paul’s Hospital, Providence Health Care site (P05–123). The study complies with the BC’s Freedom of Information and Protection of Privacy Act. The study was conducted primarily using anonymized laboratory and administrative databases, and therefore informed consent was not required. Incidence data were augmented with data collected through prospective research cohort studies, which include written informed consent by study participants and separate IRB approval.

### Role of the Funding Source

The sponsors had no role in the design, data collection, data analysis, data interpretation, or writing of the report. The corresponding author had full access to all data in the study and had final responsibility to submit for publication.

## Results

From January 1^st^ 1996 to December 31^st^ 2012, the estimated HIV prevalence in BC increased from 7,900 to 11,972 cases (52%; p-value <0.0001), and the number of individuals actively on HAART increased from 837 to 6772 (709%; p-value <0.0001). Based on these figures, we estimated that HAART coverage increased from 11% to 57% (p-value 0.0004) during this period.

### HIV Disease Progression

**Figure 1:**
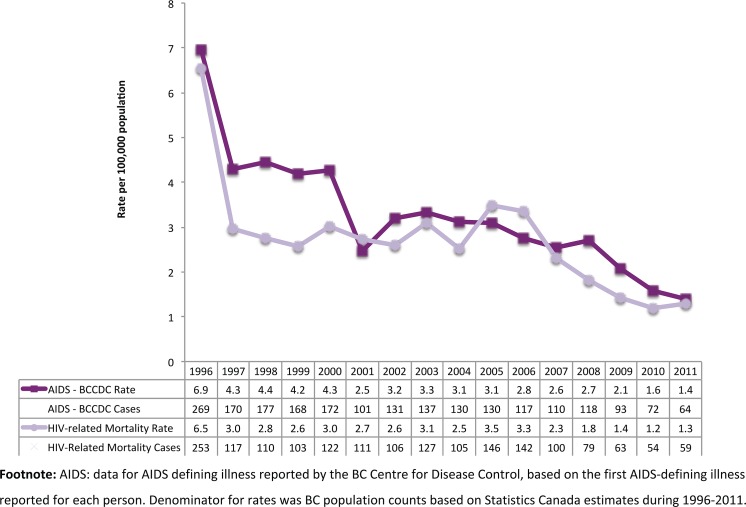
Number and rate of AIDS and death (HIV-related) cases by calendar year, 1996–2011. AIDS: data for AIDS defining illness reported by the BC Centre for Disease Control, based on the first AIDS-defining illness reported for each person. Denominator for rates was BC population counts based on Statistics Canada estimates during 1996–2011.

As shown in Figure 1, the trend in the number of HIV-related deaths from 1996 to 2011, with 253 individuals dying in 1996 and only 59 individuals dying in 2011. Using the overall BC population as a denominator, the HIV-related mortality rate decreased from 6.5 to 1.3 per 100,000 of the BC population during 1996–2011 (80% decrease, p-value 0.0115). Figure 1 also shows that the AIDS rates decreased from 6.9 to 1.4 per 100,000 population over the study period (80% decrease, p-value 0.0330).

**Figure 2:**
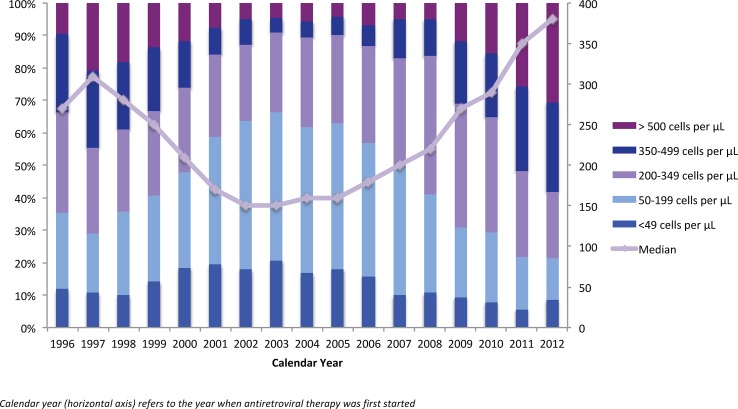
Distribution of baseline CD4 cell count by year of therapy initiation, 1996–2012. Calendar year (horizontal axis) refers to the year when antiretroviral therapy was first started.

As shown in Figure 2, the overall median baseline or pre-therapy CD4 cell count increased from 270 cells/µL (25–75^th^ percentile 130–390) in 1996 to 380 cells/µL (25–75^th^ percentile 235–550) in 2012 (41% increase; p-value <0.0001). The median pre-therapy CD4 cell count among individuals with a history of injection drug use (IDU) was 350 cells/µL (25–75^th^ percentile 180–500) in 2012.

**Figure 3:**
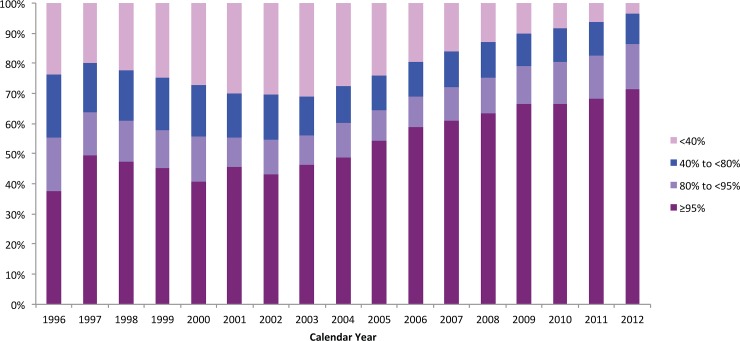
Distribution of individuals’ adherence to antiretrovirals by calendar year, 1996–2012.

As shown in Figure 3, HAART adherence levels for individuals who ever started therapy in BC increased over time. While only 37% of individuals had adherence levels ≥95% in 1996, this increased to 71% for individuals (p-value 0.0032) by 2012.

Table 1 shows the estimated aggregate viral load levels at the population-level over time. Using a 500 copies/mL cut-off, we estimated that the proportion of individuals virologically suppressed increased from 8% in 1996 to 74% in 2012 (p-value <0.0001). The analysis was repeated using a 50 copies/mL cut-off; however, this was restricted to the period of 1999–2012, as the more sensitive test was not available prior to 1999. Using a 50 copies/mL cut-off, the proportion of individuals suppressed increased from 6% to 59% (p-value <0.0001).

**Table 1:** Distribution of the highest plasma viral load from HIV infected individuals by calendar year, 1996–2012.

Year	N	Patients with <500 copies/ml (%)	Median HIV-1 RNA plasma concentration (copies per mL; Q1–Q3)	Patients with <50 copies/mL (%)	Median HIV-1 RNA plasma concentration (copies per mL; Q1–Q3)
1996	2924	224 (8%)	35500 (6800–100010)	NA (−)	NA (−)
1997	4180	585 (14%)	24000 (3200–100010)	NA (−)	NA (−)
1998	4879	1292 (26%)	13000 (499–92000)	NA (−)	NA (−)
1999	5443	1755 (32%)	9470 (499–84200)	307 (6%)	9470 (359–84200)
2000	5931	2052 (35%)	9400 (499–85000)	1387 (23%)	9400 (88–85000)
2001	6461	2386 (37%)	7700 (499–80200)	1693 (26%)	7700 (49–80200)
2002	6985	2670 (38%)	10200 (499–97000)	1939 (28%)	10200 (49–97000)
2003	7437	2902 (39%)	8960 (499–86200)	2185 (29%)	8960 (49–86200)
2004	7906	3340 (42%)	5035 (499–71200)	2577 (33%)	5035 (49–71200)
2005	8277	3775 (46%)	2230 (499–61600)	3014 (36%)	2230 (49–61600)
2006	8552	4195 (49%)	699.5 (499–52350)	3431 (40%)	699.5 (49–52350)
2007	8868	4621 (52%)	499 (499–45350)	3803 (43%)	359 (49–45350)
2008	9343	5324 (57%)	499 (499–26800)	4033 (43%)	136 (49–26800)
2009	9963	6227 (63%)	499 (499–14400)	4992 (50%)	49 (49–14400)
2010	10548	7060 (67%)	499 (499–8514)	5600 (53%)	49 (49–8514)
2011	11191	7918 (71%)	499 (499–2770)	6237 (56%)	49 (49–2770)
2012	11805	8747 (74%)	499 (499–755)	7007 (59%)	49 (49–755)

Includes all individuals ever having a plasma viral load test done regardless of whether they received antiretroviral therapy.

**Figure 4:**
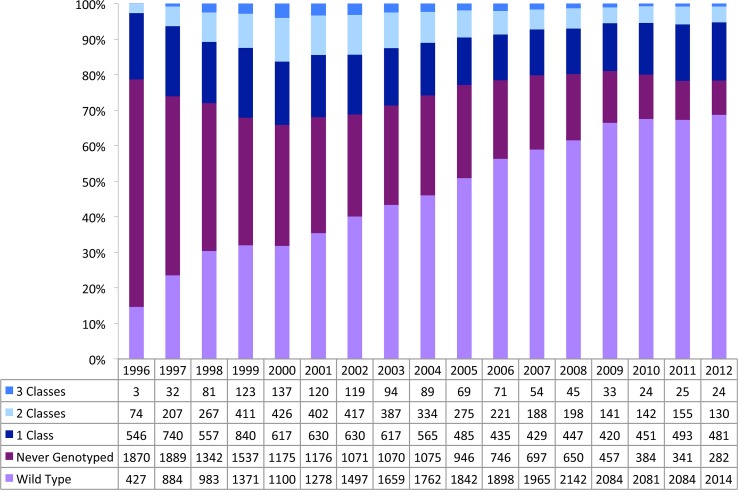
Distribution of HIV drug resistance among individuals with unsuppressed viral load by calendar year, 1996–2012.

As shown in Figure 4, emergent antiretroviral drug resistance among individuals with unsuppressed viral load (including individuals on or off therapy) decreased from 1996 to 2012 in BC. Since 1996, the number of individuals never genotyped decreased markedly from 64% in 1996 to 10% in 2012 (p-value <0.0001). At the same time, the prevalence of individuals with wild type virus (i.e.: no drug resistance) increased from 15% in 1996 to 69% in 2012 (p-value <0.0001).

### HIV New Diagnoses

**Figure 5:**
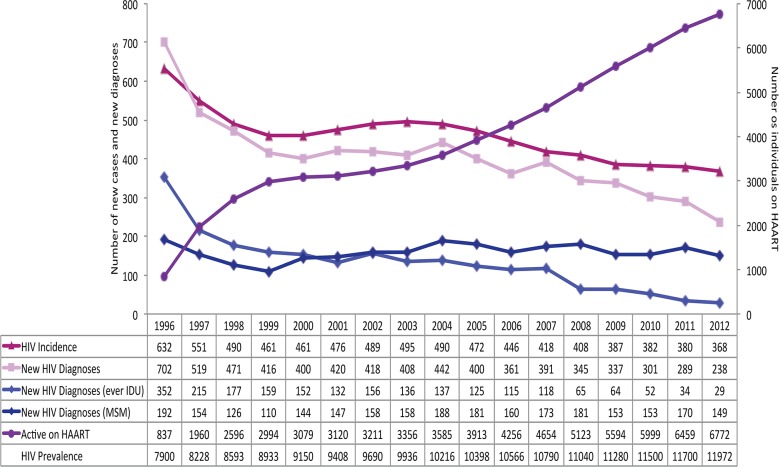
Selected HIV epidemic indicators for British Columbia by calendar year, 1996–2012.

As shown in Figure 5, there was a steady decline in the number of HIV new diagnoses from 702 to 238 cases (−66%; p-value 0.0004) and for estimated reduction of HIV incident cases from 632 to 368 cases per year (−42%; p-value 0.0003) between 1996 and 2012. New HIV diagnoses decreased by 92% (p-value 0.0013) among individuals with a history of injection drug use, and by 22% (p-value 0.0046) among MSM. Further, when the size of the MSM population was factored in, based on US CDC estimates, the rates of HIV new diagnoses was found to have declined from 4.43 per 1000 in 1996 to 3.21 per 1000 in 2004 to 1.81 per 1000 in 2012.

All confirmed HIV positive tests have been documented since 1989, however HIV became reportable in 2003 in BC with systematic follow-up of positive test results from that time forward. Therefore reports prior to 2003 are biased because duplicate positive tests were not consistently removed. All longer-term trends in Figure 5 after 2003 are statistically significant. Therefore it is unlikely that over-counting cases prior to 2003 substantially impacted the overall conclusions of the paper.

### Statistical Models

Next, we developed two statistical models with the outcome being the estimated HIV incidence rate and the explanatory variables being the number of individuals actively on HAART and the percentage of individuals suppressed on HAART (using a 500 copies/mL cut-off to allow a consistent definition of suppression over time). The model showed that for each 100 individuals actively on HAART the estimated incidence rate decreased by 1.2% (estimated rate ratio 0.9879; 95% CI 0.9868; 0.9891) and for each 1% increase in the number of individuals suppressed on HAART, the HIV incidence decreased by 1% (estimated rate ratio 0.9900; 95% CI 0.9887; 0.9912).

For the model with HIV-related mortality rate as the outcome and the explanatory variables being the number of individuals actively on HAART and the percentage of individuals suppressed on HAART (using a 500 copies/mL cut-off), we observed that for each 100 individuals actively on HAART the estimated mortality rate decreased by 2.51% (estimated rate ratio 0.9749; 95% CI 0.9703; 0.9795) and for each 1% increase in the number of individuals suppressed on HAART, the mortality rate decreased by 2.06% (estimated rate ratio 0.9794; 95% CI 0.9754; 0.9834).

For the model with the outcome being the AIDS rate and the explanatory variables being the number of individuals actively on HAART and the percentage of individuals suppressed on HAART (using a 500 copies/mL cut-off), we observed that for each 100 individuals actively on HAART the estimated AIDS rate decreased by 2.48% (estimated rate ratio 0.9752; 95% CI 0.9679; 0.9826) and for each 1% increase in the number of individuals suppressed on HAART, the AIDS rate decreased by 1.95% (estimated rate ratio 0.9805; 95% CI 0.9737; 0.9874).

## Discussion

Our results demonstrate that between 1996 and 2012, the expansion of HAART coverage in BC was strongly and statistically significantly associated with population level decreases in incident AIDS diagnoses and all cause mortality among HIV infected individuals. In addition, the expansion of HAART coverage in BC was strongly and statistically significantly associated with decreased HIV new diagnoses and estimated HIV incidence. Overall, we estimated that for every 1% increase in the number of individuals suppressed on HAART, the HIV/AIDS morbidity and mortality decreased by 2%, and HIV incidence rate decreased by 1%.

As expected, we documented a shift towards earlier initiation of HAART during the study period, as demonstrated by a steady increase in the median pre-HAART CD4 cell count. In contrast to earlier predictions based on mathematical models, [41]–[45] our results showed that overall level of adherence to HAART improved, with a consequent increase in the proportion of individuals virologically suppressed and a decrease in the prevalence of antiretroviral drug resistance during the study period. [10], [24], [46].

Of note, the changes in HIV/AIDS morbidity, mortality and transmission, took place against a background of persistently high rates of genital chlamydia and genital gonorrhea, infectious syphilis. A declining trend in the rate of hepatitis C new cases was seen during the study period and the ecological nature of our population-based study, preclude us from excluding a liberal bias in this regard. In this context, it should be noted that progressive drug policies adopted in BC, including low threshold drug treatment facilities, wide availability of needle and syringe programs, extensive opioid substitution therapy program, and the first supervised injection site in North America, have been shown to act as both facilitators of the expansion of HAART coverage among injection drug users and could have also affected HIV and HCV transmission dynamics.

Our results support the long-term population-level effectiveness and sustainability of the treatment as prevention strategy, as it relates to its impact on HIV/AIDS morbidity and mortality, as well as HIV new diagnoses, within a resource rich environment with a fully subsidized health care system. It is important to emphasize that the declining trends in new HIV diagnosed in BC are unique in Canada. [47] This is despite the fact that Canada enjoys a health care system, which is a publically funded and universally accessible. However, BC is the only jurisdiction in Canada where HIV/AIDS services, including medical services, centralized laboratory monitoring and HAART are fully subsidized, with no co-payments or deductibles. BC has also differentiated itself within Canada for the establishment of a centralized unit devoted to implementing, monitoring, and evaluating HIV/AIDS programs in the province.

It is reassuring to note that evidence in support of a population-based impact of treatment on the prevention of HIV transmission continues to emerge from diverse global settings. Such effects have been described in Taiwan, [22] and more recently in Vancouver, [9], [21], [33] Baltimore, [48] San Francisco, [23] China, [49], [50] and Kwazulu-Natal. [46] It is important to mention that the magnitude of the impact of the expansion of HAART coverage on HIV transmission derived from our models is entirely consistent with the effect noted from the experience in Kwazulu-Natal. [46] Further monitoring of the population-level impact of HAART roll out efforts will help to validate the predictions of recent [51], [52] and further characterize the field impact of TasP in diverse epidemiological situations and settings. [53] Undoubtedly, this will be greatly facilitated by the recent release of the 2013 World Health Organization (WHO) Consolidated Global HIV ARV Treatment Guidelines. [54], [55] In brief, the 2013 WHO Guidelines recommend that HAART eligibility be expanded to all HIV infected individuals regardless of symptoms if they have a CD4 cell count of less than 500/mm^3^, or regardless of CD4 count level if there is TB co-infection, or liver disease due to hepatitis B virus co-infection, or for individuals at very high risk for HIV transmission, such as sero-discordant couples and pregnant HIV infected women, who are encouraged to start HAART during pregnancy and continue lifelong therapy thereafter, and for children infected with HIV under the age of 5 years old who are also advised to continue lifelong therapy. The 2013 WHO Guidelines are expected to expand HAART eligibility to at least 80% of those infected with HIV worldwide. As such, they truly open the door for the global implementation of HIV TasP. Our data suggest that this may indeed be a game changer in the fight against HIV/AIDS, as it would be expected to dramatically curb morbidity and premature mortality, as well as HIV transmission globally, with impressive economic impact. Indeed, we have previously used mathematical modeling to project that expansion of HAART coverage, or TasP, has a favorable cost-benefit ratio when morbidity and mortality benefits are considered, and is becomes cost-averting when the HIV transmission benefit is added. [56] This has been independently confirmed by the modeling work of Granich et al [57] and, more recently by Walensky et al. [58].

As in any study of this nature, changing patterns of risk behaviours could have influenced our results. However, background incidence rates of sexually transmitted illnesses and blood borne diseases can serve as adequate surrogates for risk behaviours. In this context, it is important to note that the rates of infectious syphilis, gonorrhea, and chlamydia in BC have remained alarmingly high during the study period. Indeed, infectious syphilis is currently rising at a very high rate in BC, particularly within MSM. Specifically, over the study period in BC, infectious syphilis rate increased from 0.5 to 7.7 (1446%, p-value <0.0001), genital chlamydia rate increased from 106 to 256 (141%, p-value <0.0001), and genital gonorrhea rate increased from 14 to 30 (119%, p-value <0.0001). Indeed, a recent report by the BCCDC, [59] confirms alarmingly high and increasing rates of syphilis among MSM in BC. While there are limitations to ecological comparison (e.g., with the majority of Chlamydia infections likely occurring among younger adults due to heterosexual sex, and HIV positive MSM being over-represented among syphilis cases), against this background, the decrease in HIV new diagnoses reported here is even more striking.

In BC, over the study period, the hepatitis C rate (per 100,000 population) decreased from 172 to 36 (−79%, p-value <0.0001). As we discussed above, the latter is a reflection of the efficacy of BC’s progressive drug policies, which in turn facilitated engagement on HAART, and as such had a beneficial effect on the expansion of HAART in our setting. This is significant given persistent reluctance to offer HAART to the IDU population in some settings, often attributed to concerns regarding potential for incomplete adherence leading to HIV drug resistance and the threat of spreading primary HIV drug resistance to the population at large, [60] which may in turn compromise the effectiveness of the TasP, as recently suggested. [43] Also, we should acknowledge that there has been some reluctance to embrace TasP within IDU communities as they have (rightly so, in some settings) advanced the argument that TasP could compete for resources against other valuable evidence based initiatives, particularly harm reduction, and that TasP may be used as a pretext to promote coercive testing and treatment initiatives. These are sad realities that need to be overcome if the full benefit of TasP is to be globally realized. Further, given that IDU continue to drive new infections in several settings contending with growing HIV epidemics, [61] such as Russia, and given that IDU have high potential to promote more generalized epidemics of HIV infection, [61] the data presented herein suggest that there is an urgent need to ensure that TasP initiatives are extended to IDU on a global scale without coercion and with continued promotion of other evidence based interventions, including harm reduction.

Our report is derived from a population based ecological study within the province of BC, in Canada. As a result, we cannot draw a causal link between increased HAART coverage and declines in HIV/AIDS morbidity, mortality, and HIV transmission. With regard to clinical outcomes, we cannot find an alternative explanation or confounder that may explain the association between HAART coverage expansion and declining HIV/AIDS morbidity and mortality uncovered by our models. With regard to transmission, we needed to account for changes in risk behaviours as a potential confounder, as discussed above. As described above, the high and increasing rates of STIs during the study period suggest that high prevalence of risky sexual behaviours over the study period. In contrast, the declining trends in HCV diagnoses suggest that high-risk injection practices were on the decline during the study period. These findings suggest that there may be a conservative bias in our estimates for sexual transmission, and a liberal bias for injection based transmission. Further work will be needed to derive more precise estimates of the degree of protection that HAART offers in each setting, and the relative contribution of specific risk factor modification strategies. However, we are reassured by the similarity of our overall estimates for the impact of the expansion of HAART coverage on HIV transmission between the present study and a recently reported population based study from Kwazulu-Natal, in South Africa. [46] Additionally, our findings are entirely consistent with those of HPTN 052, [10] a recently reported randomized clinical trial that demonstrated that immediate ART decreased sexual HIV transmission by 96.3% among sero-discordant heterosexual couples.

In conclusion, our results show that HAART expansion in BC between 1996 and 2012 was strongly and statistically significantly associated with sustained population-level decreases in HIV/AIDS related morbidity and mortality, as well as concomitant decreases in new HIV diagnoses, and estimated HIV incidence. Expansion of HAART coverage was also associated with increased adherence rates, increased rates of viral suppression, and decreased rates of HIV resistance. Overall, our model suggests that for every 1% increase in the number of individuals suppressed on HAART, the estimated HIV incidence also decreased by 1%. Our results support the real world and long-term effectiveness and sustainability of the HIV treatment as prevention strategy, within a resource rich environment with a fully subsidized health care system. The 2013 Consolidated World Health Organization Antiretroviral Therapy Guidelines offer a unique opportunity to further evaluate this issue in other settings, particularly within generalized epidemics, and resource-limited setting, within the context of much needed programmatic HAART expansion initiatives, as advocated by UNAIDS.
